# Effectiveness rehabilitative therapy and Pridinol Mesylate in low back pain

**DOI:** 10.3389/fmed.2024.1470996

**Published:** 2025-01-22

**Authors:** Lorenza Lauricella, Noemi Calabrese, Dalila Scaturro, Domenico Migliorino, Michele Vecchio, Giulia Letizia Mauro

**Affiliations:** ^1^Paolo Giaccone University Hospital Policlinico, Palermo, Italy; ^2^University of Catania, Catania, Italy; ^3^Department of Surgical, Oncological and Stomatological Disciplines, University of Palermo, Palermo, Italy; ^4^Section of Pharmacology, Department of Biomedical and Biotechnological Sciences, University of Catania, Catania, Italy

**Keywords:** Pridinol, low back pain, postural balance, exercise, rehabilitation

## Abstract

**Background:**

Spondylarthritis is a degenerative disease involving the intervertebral disc, vertebral bodies, and adjacent soft tissues. Treatment aims to slow disease progression and manage symptoms through an interdisciplinary approach. It can be conservative and rarely chirurgic.

**Objective:**

This study aimed to evaluate the effectiveness of a rehabilitation combined with Pridinol Mesylate in the treatment of Spondylarthritis in elderly patients in terms of pain resolution, improving disability, and quality of life.

**Materials and methods:**

We conducted a retrospective study on patients with Spondylarthritis. The patients recruited (*n* = 86) were divided into three groups: the Combined Group (CG:28), who received a rehabilitation combined with Pridinol Mesylate (16 women and 12 men, age 66.4 ± 3.99); the Rehabilitation Group (RG, *n* = 26), who received only rehabilitation (14 women and 12 men, age 66.2 ± 3.84); and the Drug Group (DG: 32), who received only the administration of the Pridinol(18 women and 14 men, age of 66.3 ± 3.9).

**Results:**

The results show, at T1 (20 days after treatment) in the CG, statistically significant improvements for the NRS and QBPDS. In the RG, statistically significant improvements were observed only for the QBPDS scale. In the DG group, only pain improvement. At T2 (90 days after treatment), the CG showed improvements in NRS, QBPDS, and (SF-36). The RG and DG showed improvements for NRS and for QBPDS. By Bonferroni method, obtained statistically significant values for CG versus RG and for CG versus DG. No statistical significance was found between RG versus DG.

**Conclusion:**

Targeted rehabilitation treatment, combined with Pridinol Mesylate, reduced pain and improved disability in lumbar Spondylarthritis both in the short and medium term, with improved quality of life in elderly patients.

## 1 Introduction

The spine supports and protects the spinal cord and nerve endings located in the spine. The spine is essential for maintaining good static and dynamic posture. Spondylarthritis is a degenerative disease involving the intervertebral disc, vertebral bodies, and adjacent soft tissues (1). It can affect all areas of the spine and in most cases affects the lumbar region (2).The lumbar spine exhibits lordosis in the sagittal plane and is generally subjected to severe stress. Wear and tear of the spine results in loss of stability of the structure, abnormal stresses with altered loads, and consequently localized pain in the affected region (3). Cartilage degeneration secondary to wear and aging can result in the formation of bony spines, marginal to the vertebral bodies, called spondylophytes (4). They can fuse and alter the shape of the spine, resulting in pain in the spine with possible irradiation to the lower limbs if there is radicular compression, muscle contractures, stiffness, and functional limitation (5).

Treatment aims to slow disease progression and manage symptoms through an interdisciplinary approach involving a collaborative process between different professionals: general practitioner, physiatrist, physiotherapist, rheumatologist, and orthopedist. Conservative treatment of Spondylarthritis primarily involves lifestyle changes in the early stages of the disease that can slow progression; reduction of body weight; reduction of loading activities; healthy diet; regular physical activity; therapeutic exercise and physical therapy; drug therapy; and spinal orthoses.

According to the EULAR 2018 recommendations, exercise, pilates, and massage therapy are recommended in people with Spondylarthritis (6, 7).

The most recent recommendations include the use of NSAIDs, COX-2, and muscle relaxants, which are effective on both pain and mobility (5). The interventional approach involves infiltrative treatment with hyaluronic acid, oxygen-ozone, PRP, and stem cells as well as CT-guided radiofrequency application. Surgical intervention is considered only in extreme cases, that is, when: neurological signs appear, symptoms are persistent, and all conservative treatments have been exhausted.

The muscle relaxants represent a heterogeneous group of drugs used mainly for two clinical conditions: spasticity and muscle contracture. They can be divided into peripherally acting muscle relaxants, which reduce excitation-contraction coupling, and centrally acting muscle relaxants, which reduce polysynaptic reflexes. Some of these muscle relaxants are included in the category of minor tranquilizers. The major drugs on the market are Baclofen, Cyclobenzapine, Diazepam, Eperisone, Tizanidine, Thiocolchicoside, Dantrolene, Carisoprolol and Pridinol Mesilate (8, 9).

There are several types of muscle relaxants including Pridinol Mesylate, a centrally-acting muscle relaxant that reduces polysynaptic reflexes through an anticholinergic mechanism (10). It is a piperidinpropyl alcohol derivative whose chemical formula is as follows: 1,1-diphenyl-1-ol-3-piperidino-propane methanesulfonate. Its pharmacological action is carried out by an atropine-like mechanism. According to drug characteristics, pridinol reaches maximum plasma concentration within 1 h after oral administration and is evenly distributed in tissues (10). It is mainly metabolized through cytochrome P450 (CYP)2C19 and CYP2B6 into its major metabolite 4-hydroxypridinol (11). It is eliminated renally partly as an unchanged drug and partly as a glucuronate or sulfoconjugate drug (12). It is indicated in the treatment of skeletal muscle contractures, whether of central or peripheral origin. The myorelaxant effect of Pridinol Mesylate is at the level of both smooth and striated muscle tissue, owing to its action as an antagonist of the muscarinic acetylcholine receptor and inhibition of stimulus conduction in spinal motor neurons. The possible side effects of Pridinol Mesylate are: tachycardia, drowsiness, hypotension, nausea and abdominal pain, asthenia, headache and diarrhea (13, 14).

Literature has demonstrated the effect of Pridinol used alone (9) and the effectiveness of rehabilitation treatment (5). However, no one has used the combination and demonstrated their synergy of action in pain management and patient quality of life.

## 2 Objective

This study aimed to evaluate the effectiveness of a rehabilitation program combined with Pridinol Mesylate in the treatment of Spondylarthritis in elderly patients in terms of pain resolution, improving disability, and quality of life versus single treatment.

## 3 Materials and methods

### 3.1 Trial design

We conducted retrospecitive study on outpatients who attended the rehabilitation clinic of the University Hospital of Palermo for Spondylarthritis. The study period was between March 2022 and June 2024. The study received approval from the local ethical committee “Palermo 1” (approval no. 05/2023) and was conducted by the declaration of Helsinki. The processing of information and data has been carried out according to the guidelines of Good Clinical Practice (GCP). All participants gave their written informed consent.

### 3.2 Participants

The inclusion criteria were: age 65–75 years, low back pain for at least 3 months, NRS ≥ 4 (average pain rating on the day of recruitment), radiographic diagnosis of Spondylarthritis (15), and written informed consent. Patients were excluded in case of inflammatory diseases of the spine, obesity (BMI >30), positive radicular tests, allergy, or contraindications to Pridinol Mesylate.

### 3.3 Intervention

We extracted 546 patients diagnosed with lumbar Spondylarthritis from our hospital database. Of these, only 91 met the inclusion criteria and therefore were included in the study. Subsequently, 5 patients were excluded because they did not present for follow-up. None of the patients discontinued the proposed treatment, although drug therapy with Pridinol Mesylate caused self-resolving diarrhea and drowsiness in 2 patients. The 86 patients recruited were divided into three groups: the Combined Group (CG), consisting of 28 patients, who received a rehabilitation program combined with the administration of Pridinol Mesylate 4 mg (1/2 tablet 3 times a day for 20 days); the Rehabilitation Group (RG), consisting of 26 patients, who received only the same rehabilitation program; and the Drug Group (DG), consisting of 32 patients, who received only the administration of the drug Pridinol Mesylate 4 mg (1/2 tablet 3 times a day for 20 days).

### 3.4 Outcomes

During the initial clinical assessment, demographic (age, sex, BMI) and clinical information was collected. For each clinical assessment, rating scales were administered by the same physiatrist, such as the Numeric Rating Scale (NRS) to assess the extent of pain; the Quebec Back Pain Disability Scale, to provide a reliable assessment of low back pain-related disability; SF-36, to assess the patient’s quality of life. All this information was assessed at 3 times: enrollment (T0), end of treatment (T1), and 90 days after the start of treatment (T2).

#### 3.4.1 Rehabilitation program

The rehabilitation included daily sessions, 5 days a week, lasting 60 min, for a total of 20 sessions. The planned rehabilitation was performed under the supervision of a therapist. The intensity and volume of the rehabilitation treatment were established, following the recommendations of the American College of Sports Medicine (16–18).

Participants had access to the rehabilitation gymnasium in our department and were asked to come with sportswear and a neoprene mat to perform the same rehabilitation project-program.

Each session involved a 1:1 ratio of patient to physical therapist, with decades of experience in Postural Reeducation. It was explained to the patient to always perform the exercises on a hard surface, with slow movements, and with programmed breathing acts. The treatment included an initial step, lasting 40 min, of Postural Reeducation ending with Stretching exercises of the posterior kinetic chain muscles lasting 20 min (19–21).

Postural re-education included: calisthenic exercises, mobilization of the upper limbs first and then of the lower limbs, proprioceptive exercises and muscle strengthening exercises. The exercises, carried out with the help of the physiotherapist, were: Dead bug (3 sets), Hallow position (4 sets), Prone chest raise (3 sets), Side plank (3 sets), Isometric Pallof Press (3 sets), Dynamic Pallof Press (3 sets), Box squat (4 sets), Lunges with pad (4 sets) and Single foot bridge (4 sets).

#### 3.4.2 Drug posology

The Supplement Group had taken Pridinol Mesylate orally, on an empty stomach, 2 mg x 3 times a day. The tablet was taken without chewing and with a glass of water (200 mL). The drug was taken in environments with temperatures below 25° C. (8, 18).

#### 3.4.3 Rating scales

The NRS is a one-dimensional 11-point scale that rates pain intensity in adults. The scale consists of a horizontal line with a numerical range from 0 to 10, corresponding to “no pain” and “worst pain imaginable,” respectively. The patient indicates the intensity of his or her pain verbally or by drawing a circle on the number that best describes it (9).

The Quebec Back Pain Disability Scale (QBPDS) is a 20-item rating scale that measures an individual’s ability to perform various daily activities, including walking, sitting, lifting objects, and bending. Each item has 6 possible responses, with scores from 0 to 5, where 0 indicates no difficulty and 5 indicates severe disability (10, 11).

The SF-36 is a questionnaire comprising eight multiple-choice questions that can be divided into two large subgroups: the physical component of the disease and the mental component of the disease. A score is assigned to each scale; the higher the score, the better the state of mental and physical health. The score ranges from 0 (worst state of health) to 100 (best state of health). The MCID for this scale is 4.9 points (22).

### 3.5 Statistical analysis

Data collection was carried out through the use of a spreadsheet (Microsoft Excel, version 16.58). Through the use of the Shapiro–Wilk test, the normality of the collected data was checked. The text and tables report continuous variables, expressed as means, standard deviations and categorical variables, expressed as absolute numbers. For statistical analysis of the data, the *t*-test was used to compare averages among quantitative variables, while the Mood median test was used to compare medians among categorical variables. Results showing *p* ≤ 0.05 were considered statistically significant. Finally, the groups were compared using the Bonferroni method.

## 4 Results

From the analysis of the results, we observed that the three groups at T0 had homogeneous demographic and clinical characteristics as summarized in Table 1. Participants were mainly women (51.16%), with an average age of 66.4 ± 3.96 years and an average BMI of 25.6 ± 2.8 kg/m^2^. 20 patients had a primary school diploma, 38 patients had a secondary school diploma, and 28 patients had a university degree. More than half of the participants (*n* = 45) were not smokers. The average daily working hours of the recruited sample were 9.6 ± 3.2 h. The mean perceived pain was 6.24 ± 0.82 points according to the NRS scale, with a mean score on the QBPDS scale of 34.3 ± 1.81 and the SF-36 scale of 64.52 ± 2.36. There were no significant differences between the participants of the three study groups regarding the different baseline characteristics analyzed. Among the subjects recruited for the study, 29.24% had comorbidities (Table 1).

**Table 1:** General patient characteristics.

Characteristics	Total (*n* = 86)	CG (*n* = 28)	RG (*n* = 26)	DG (*n* = 32)	*p*-value
Age, mean SD	66.4 ± 3.96	66.4 ± 3.99	66.2 ± 3.84	66.3 ± 3.9	0.64
Sex, no.					
Male	44	16	14	14	
Female	42	12	12	18	
BMI, mean ± SD	25.6 ± 2.8	25.8 ± 2.1	26.3 ± 2.2	25.6 ± 3.2	0.13
Primary school	20	12	5	3	0.56
Secondary school	38	15	13	10	
Degree	28	7	10	13	
Smoker no.					
Yes	41	10	14	17	0.43
No	45	18	12	15	
Workhours, mean ± SD	9.6 ± 3.2	9.9 ± 3.1	9.5 ± 2.9	9.2 ± 2.8	0.53
NRS, mean ± SD	6.20 ± 0.82	6.24 ± 0.91	6.17 ± 0.72	6.23 ± 0.61	0.11
QBPDS, mean ± SD	32.31 ± 1.81	34.32 ± 1.52	33.03 ± 1.91	34.51 ± 1.68	0.5
SF36, mean ± SD	60.52 ± 2.36	64.52 ± 5.87	57.81 ± 6.97	55.82 ± 7.73	0.34
Comorbidity	34 (29.24%)	14 (12.04%)	11 (9.46%)	9 (7.74%)	

Table 2 shows the changes in the variables examined in the three groups at T1. In the CG, we observed statistically significant improvements for NRS scale (T0 6.24 ± 0.91 and T1 3.56 ± 0.33, *p*-value <0.05) and for QBPDS scale (T0 34.32 ± 1.52 and T1 28.18 ± 3.75, *p*-value <0.05). In the RG, statistically significant improvements were observed only for QBPDS scale (T0 33.03 ± 1.91 and T1 29.82 ± 4.15, *p*-value <0.05). In the DG, only pain showed statistically significant improvements after treatment for pain reduction (NRS: T0 6.23 ± 0.61 and T1 3.94 ± 0.58, *p*-value <0.05).

**Table 2:** Effects of the different treatments in the CG, in the RG, and in the DG at T1.

Characteristics	CG	RG	DG
NRS, mean ± SD			
T0	6.24 ± 0.91	6.17 ± 0.72	6.23 ± 0.61
T1	3.56 ± 0.33	5.91 ± 0.41	3.94 ± 0.58
*p*-value	<0.05	0.13	<0.05
QBPDS, mean ± SD			
T0	34.3 ± 1.52	33.03 ± 1.91	34.51 ± 1.68
T1	28.18 ± 3.75	29.82 ± 4.15	34.34 ± 3.97
*p*-value	<0.05	<0.05	0.81
SF-36,mean ± SD			
T0	64.52 ± 5.82	57.81 ± 6.92	55.82 ± 7.73
T1	65.22 ± 4.36	58.24 ± 7.23	56.48 ± 6.51
*p*-value	0.19	0.12	0.48

Table 3 shows the changes in the variables examined in the three groups at T2. The CG showed statistically significant improvements for NRS scale (T0 6.24 ± 0.91 and T2 3.16 ± 0.42, *p*-value <0.05) and for QBPDS scale (T0 34.32 ± 1.52 and T2 20.15 ± 3.99, *p*-value <0.05), and for SF-36 (T0 64.52 ± 5.82 and T2 81.66 ± 6.24, *p*-value <0.05). The RG showed statistically significant improvements for NRS (T0 6.17 ± 0.72 and T2 3.23 ± 0.61, *p*-value <0.05) and for QBPDS scale (T0 33.03 ± 1.91 and T2 26.37 ± 3.92, *p*-value <0.05). A statistically significant improvement was also observed in the DG for NRS (NRS: T0 6.23 ± 0.61 and T2 4.67 ± 0.33, *p*-value <0.05) and QBPDS (T0 34.51 ± 1.68 and T2 28.23 ± 4.95, *p*-value <0.05).

**Table 3:** Effects of the different treatments in the CG, in the RG, and in the DG at T2.

Characteristics	CG	RG	DG
NRS, mean ± SD			
T0	6.24 ± 0.91	6.17 ± 0.72	6.23 ± 0.61
T2	3.16 ± 0.42	3.23 ± 0.61	4.67 ± 0.33
*p*-value	<0.05	<0.05	<0.05
QBPDS, mean ± SD			
T0	34.32 ± 1.52	33.03 ± 1.91	34.51 ± 1.68
T2	20.15 ± 3.99	26.37 ± 3.92	28.23 ± 4.95
*p*-value	<0.05	<0.05	<0.05
SF-36,mean ± SD			
T0	64,52 ± 5.82	57.81 ± 6.92	55.82 ± 7.73
T2	81.66 ± 6.24	59.53 ± 8.22	58.18 ± 6.26
*p*-value	<0.05	0.02	0.09

Table 4 shows the comparison between the results obtained in the three groups in T1 and T2. At T1, the CG showed statistically superior results compared to the other groups only in terms of pain *p*-value (<0.05) and disability (<0.05). No statistically significant differences were present between the three groups at T1 for quality of life. At T2, the CG showed statistically superior results compared to the other groups regarding pain (*p* < 0.05), disability (*p* < 0.05), and quality of life (*p* < 0.05).

**Table 4:** Comparison T1/ T2 between CG-RG-DG.

Characteristics	T1				T2			
	CG	RG	DG	*p*-value	CG	RG	DG	*p*-value
NRS, mean ± SD	3.56 ± 0.33	5.91 ± 0.41	3.94 ± 0.58	<0.05	3.16±0.42	3.23 ± 0.61	4.67 ± 0.33	<0.05
QBPDS, mean ± SD	28.18 ± 3.75	29.82 ± 4.15	34.34 ± 3,97	<0.05	20.15 ± 3.99	26.37 ± 3.92	28.23 ± 4.95	<0.05
SF-36, mean ± SD	65.22 ± 4.36	58.24 ± 7.23	56.48 ± 6.51	0.18	81.66 ± 6.24	59.53 ± 8.22	58.18 ± 6.26	<0.05

GLM procedure was calculated by Bonferroni method, then we performed t-tests for each group and obtained statistically significant values for CG versus RG (*p*-value 0.0363) and for CG versus DG (*p*-value 0.0114). No statistical significance was found between RG versus DG (Table 5).

**Table 5:** Bonferroni method comparison between CG-RG-DG.

	*p*-value
CG vs RG	*p* = 0.0363*
CG vs DG	*p* = 0.0114*
RG vs DG	*p* = 0.1785

*Indicates statistical significance.

## 5 Discussion

Spondylarthritis is a degenerative disease of the spine, and treatment aims to slow the progression of the disease, manage symptoms, and prevent flare-ups (2). The high frequency of this pathology has a notable impact from both a clinical and economic point of view, for this reason the scientific literature provides numerous studies on the subject, but nevertheless the data collected is not uniform mainly due to the proposed treatment. Conservative therapy is always proposed as the first choice treatment.

In this study, we evaluated the effectiveness of rehabilitation treatment (postural re-education and stretching) combined with the administration of Pridinol Mesylate, a centrally acting muscle relaxant, in the treatment of Spondylarthritis in elderly patients compared to single treatments. Efficacy was evaluated in terms of pain resolution, disability and quality of life (8).

In line with our findings, Kim B. et al. (23) proposed a program of core stability exercises and hip muscle stretching for the treatment of patients with spondyloarthritis, demonstrating that postural re-education and hip muscle stretching are effective in improving physical function and physical activity compared to massage therapy and stretching exercises alone. Postural re-education was also proposed by Waongenngarm P. et al. (13) as a treatment for patients with low back pain, effective for preventing new onset of low back pain during a 6-month follow-up among high-risk employees. 65% of patients show comorbidities such as hypertension, type 2 diabetes mellitus and osteoporosis. Gębska M. et al. proposed therapeutic exercise as the only treatment for pain caused by muscle contracture, evaluating its effectiveness in female patients. They observed that therapeutic exercise is a simple and safe intervention that improves pain in these patients (24). Bodes Pardo G. et al. conducted a single-blind, randomized controlled trial on an effective educational program for chronic low back pain. 73% of patients show comorbidities such as arthrosis and hypertension (25). Grazio S. et al. (26) added that therapeutic exercise reduces disability and severity of pain, improving the status of patients with chronic low back pain and decreasing the rate of recurrence. They noted that individual, supervised exercise programs are associated with the best outcomes. Hernando-Jorge A. et al. (27) have compared the effectiveness of different types of therapeutic exercise in people with chronic spinal pain, demonstrating that there is no superior therapeutic exercise modality, but that the combination of different therapeutic exercise modalities is the complete tool for spinal pain management chronic. Finally, a meta-analysis by Fernández-Rodríguez demonstrated that therapeutic exercise reduces pain and disability in patients with chronic spondyloarthritis. They included 118 studies and recruited 9,710 participants, finding that therapeutic exercise reduced pain in 93% of cases and disability in 98% of cases. The most beneficial programs were those that included at least 1 or 2 sessions per week, with sessions of less than 60 min, and exercise programs of 3–9 weeks (28–30).

To the best of our knowledge, our study is the first to evaluate the combination of Pridinol Mesylate with therapeutic exercise compared to individual treatments.

Pridinol mesylate is a non-benzodiazepine antispasmodic indicated for muscle pain; this is demonstrated by a meta-analysis conducted by Überall et al. in which they point out that it is effective in reducing stiffness and limitation of movement. It is a drug with a high safety profile and overall therapeutic efficacy compared with placebo (14).

Another retrospective study, conducted by the same author et al. on 666 patients with musculoskeletal pain, showed that Pridinol mesylate is well tolerated and effective in reducing both pain, pain medication intake and disability, with improved quality of life in agreement with our results (9).

Other authors have evaluated the effect of other muscle relaxants in the treatment of low back pain using other drugs (muscle relaxants and anti-inflammatory drugs). For example, Tüzün F. et al. evaluated the efficacy of intramuscular injection of Thiocolchicoside (4 mg–2 mL) compared with placebo administered twice daily for 5 days in patients with acute low back pain. They conducted a multicenter, randomized, double-blind, placebo-controlled study. Hospitalized patients with acute low back pain (one hundred forty-nine) were included. Both groups showed improvement in spontaneous pain assessed by VAS at the end of day 1; however, improvement was statistically significant in the thiocolchicoside group at day 3. Hand-to-floor distance and muscle spasm decreased significantly at day 5 in the Thiocolchicoside group. The researchers therefore concluded that twice-daily administration of 4 mg of Thiocolchicoside for 5 days provides an effective and safe treatment for patients with acute low back pain accompanied by muscle spasm (31).

Karmakar A. et al. compared the efficacy and safety of two available fixed-dose combinations (FDCs), i.e., a double FDC (DFC) of etoricoxib (60 mg) and thiocolchicoside (4 mg) versus a triple FDC (TFC) of chlorzoxazone (500 mg), diclofenac (50 mg), and acetaminophen (325 mg) for 28 days. They included 200 eligible adult subjects aged 18–70 years with a history of Low Back Pain and muscle spasms for ≤14 days and Wong-Baker facial pain score > 4. They found a significant decrease in pain intensity and significant improvement in functional ability after treatment with DFC or TFC. They concluded that both DFC and TFC were comparable in terms of efficacy and safety for the management of recent-onset LBP. However, significantly more subjects with very severe pain or functional disability showed improvement after 28 days when treated with DFC compared with TFC (32).

We compared this manuscript with that of JL. et al. related to low back pain in the elderly. They conducted a review, evaluating the evidence for the effectiveness of drugs used for spine pain in the elderly, with special attention to drug metabolism and adverse drug reactions. The following drugs were reviewed: nonsteroidal anti-inflammatory drugs (NSAIDs), acetaminophen, corticosteroids, gabapentin and pregabalin, anti-spastic and antispastic muscle relaxants, tricyclic antidepressants (TCAs), serotonin-norepinephrine reuptake inhibitors (SNRIs), tramadol, and opioids. The review covered a total of 138 double-blind, placebo-controlled studies. Strong evidence emerged for the use of NSAIDs. Gabapentin and pregabalin obtained good evidence for neuropathic pain, NSAIDs and acetaminophen for arthritic and myofascial pain, and antispastics for myofascial pain (33).

In contrast to this manuscript, van Tulder MW et al. proposed cyclobenzaprine as a treatment and obtained an overall improvement in symptoms by the 10th day of treatment.

The muscle relaxant cyclobenzaprine is a useful drug for low back pain, used to reduce muscle spasms and control pain. There are several reviews on this topic, such as one by Cochrane that groups non-benzodiazepine drugs; a second serves as a starting point for a joint technical guideline by the American Pain Society and the American College of Physicians; and the third is a comprehensive review of cyclobenzaprine by a group of independent authors. The latter review examined 14 publications, comparing cyclobenzaprine with placebo, and was used as the basis for the joint clinical guideline. Adverse effects of cyclobenzaprine were drowsiness, dry mouth, dizziness and nausea, which occurred in 53 percent of participants. Improvements in low back pain symptoms were present at all recorded time points, but the authors noted a greater effect in the first 4 days of treatment. 78% of patients show comorbidities such as arthrosis and hypertension (28).

From our results, we observed that rehabilitative treatment combined with the administration of Pridinol Mesylate showed statistically superior improvements in terms of reduction of pain and disability related to low back pain in the short term; the result was maintained in the medium term, with significant improvement in quality of life also observed in the latter case. The novelty of our study was to demonstrate how this synergy (muscle relaxant and therapeutic exercise) is effective in patients with chronic lumbar Spondylarthritis.

### 5.1 The study’s limitations

The main limitations of our study are the small sample size, which limits the generalizability of the study results, the lack of follow-up at a distance, and the “retrospective” study design. Therefore, further research should be conducted with randomized clinical trials on a larger number of patients.

## 6 Conclusion

This study showed that rehabilitation combined with Pridinol Mesylate reduced pain and improved disability in lumbar Spondylarthritis both in the short and medium term, with improved quality of life in elderly patients. Therefore, we could assume that the combination of Physiotherapy and Pridinol Mesylate is a beneficial therapeutic strategy in the management of lumbar Spondylarthritis pain. We will continue this experimental study to extend it to a larger sample of patients and expand the results obtained.

## Data Availability

The datasets presented in this study can be found in online repositories. The names of the repository/repositories and accession number(s) can be found in the article/supplementary material.
